# Fatal Case of Liver and Brain Abscesses Due to Fusobacterium nucleatum

**DOI:** 10.7759/cureus.19671

**Published:** 2021-11-17

**Authors:** Nadia Toumeh, Megha Mudireddy, Bradley Smith, Dubert M Guerrero

**Affiliations:** 1 Internal Medicine, University of North Dakota School of Medicine and Health Sciences, Fargo, USA; 2 Infectious Diseases, Sanford Health, Fargo, USA; 3 Internal Medicine, University of North Dakota, Fargo, USA

**Keywords:** fatal outcome, odontogenic infection, brain abscess, liver abscess, fusobacterium

## Abstract

*Fusobacterium nucleatum *is an anaerobic gram-negative organism regarded as an oral commensal. We present a case of a 63-year-old male presenting with weakness, encephalopathy and right upper quadrant palpable mass found to have *F. nucleatum* liver abscess with innumerable intracranial abscesses. *F. nucleatum* is a rare cause of concomitant liver and brain abscesses associated with odontogenic infection with potentially fatal outcomes.

## Introduction

*Fusobacterium* is an anaerobic gram-negative bacillus that encompasses approximately 13 different species, with most human pathology being caused by *Fusobacterium necrophorum* and *Fusobacterium nucleatum*. The former is commonly implicated in Lemierre’s disease, an acute jugular vein septic thrombophlebitis often preceded by severe infections of the head and neck. On the other hand, *F. nucleatum* can be found as a commensal organism in an oropharyngeal cavity, as well as a component of the genital, gastrointestinal, and upper respiratory tracts. It is often implicated in pleuropulmonary, obstetric infections, brain and intra-abdominal abscesses [1,2].

Fusobacterial pyogenic liver abscesses have risk factors that include malignancy, dialysis treatment, and advanced age but have also been reported in immunocompetent individuals who have recently been exposed to factors that allow for hematogenous spread such as periodontal disease and pharyngitis [3,4]. Additionally, brain abscesses caused by *Fusobacterium* spp. are uncommon accounting for about 6% of cases in one report. Most cryptogenic cases are thought to be due to dental infections as well [5]. Here, we describe a rare fatal case of *F. nucleatum* infection of presumed odontogenic origin complicated by brain and liver abscesses in a patient with no known immunosuppression.

## Case presentation

Here we report a case of a 63-year-old male with alcohol and tobacco use disorder who was found on the floor of his residence due to generalized weakness. Upon admission, the patient was lethargic and unable to provide a meaningful history. Initial vital signs were stable with temperature of 99.3 F, heart rate 86 bpm, blood pressure 178/92 mmHg, and respiratory rate of 18 breaths/minute on room air. Initial laboratory findings are summarized in Table 1 significant for leukocytosis, elevated inflammatory markers and transaminases.

**Table 1:** Laboratory findings at admission RBC: red blood cell; WBC: white blood cell; BUN: blood urea nitrogen; ALP: alkaline phosphatase; ALT: alanine aminotransferase; AST: aspartate aminotransferase; CRP: C-reactive protein

	Admission	Reference range
Hemoglobin	14.8	13-15 g/dL
RBC	4.51	4.6-6.8 x 10^6^/mcL
WBC	19.3	3.6-10.3 x 10^3^/mcL
Platelet	409	140-420 x 10^3^/mcL
Absolute neutrophils	16	1.8-8.0 K/µL
Absolute lymphocytes	1.7	0.8-4.1 K/µL
Blood glucose	144	70-100 mg/dL
Sodium	142	135-145 mmol/L
Potassium	4.0	3.7-5.1 mmol/L
Chloride	104	96-110 mmol/L
Bicarbonate	29	22-32 mmol/L
BUN	25	6-24 mg/dL
Creatinine	0.71	0.6-1.3 mg/dL
Calcium	8.7	8.5-10.5 mg/dL
Bilirubin total	2.2	0.2-1.2 mg/dL
ALP	137	30-150 U/L
ALT	252	0-35 U/L
AST	167	0-35 U/L
CRP	116	0.0-8.0 mg/L
Lactic acid	2.3	0.5-2.2 mmol/L

**Figure 1:**
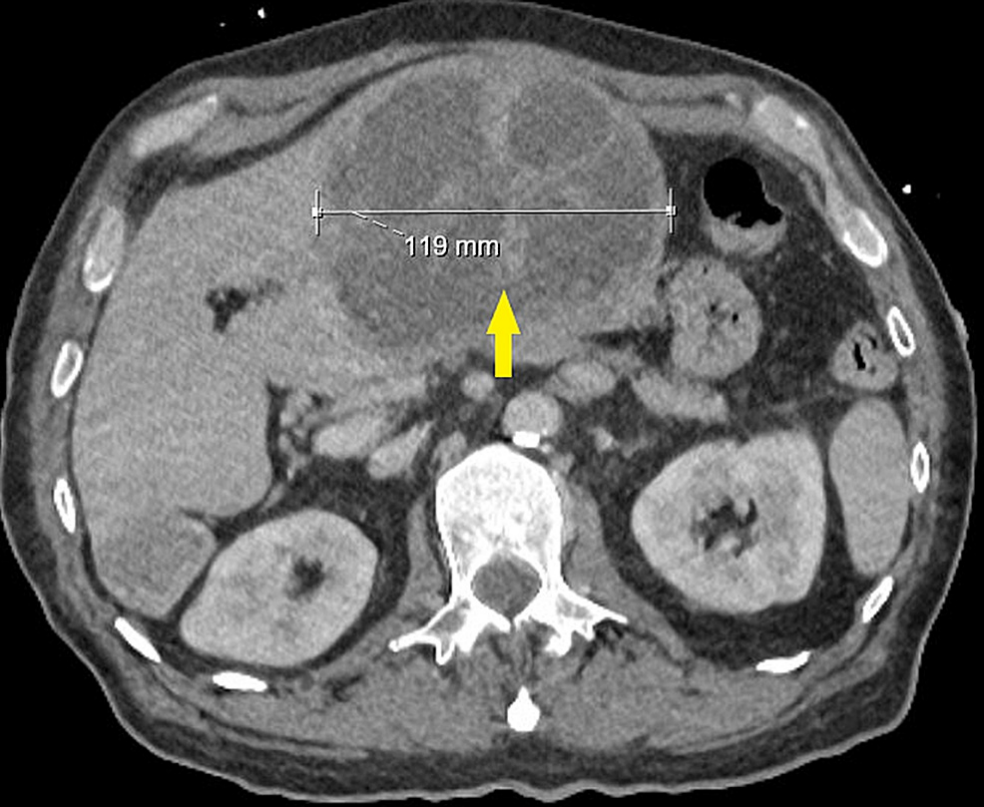
Computed tomography of abdomen showing large multiloculated liver abscess

Physical examination was notable for the patient appearing disheveled with notes of poor dentition and multiple dental caries. He had some right upper quadrant tenderness and mass presence was noted on physical examination. An ultrasound of the abdomen showed a large heterogeneous lesion in the left hepatic lobe with lobular contours and several foci of hyperechoic echogenicity and posterior acoustic shadowing. Abdominal computed tomography (CT) scan was also done with a note of the large liver abscess (Figure 1). An ultrasound-guided percutaneous drain of the left hepatic abscess was performed and 500 mL of purulent serosanguinous fluid was removed. This drainage grew* F. nucleatum* and the patient was treated with piperacillin-tazobactam for antimicrobial coverage. Blood cultures remained no growth.

**Figure 2:**
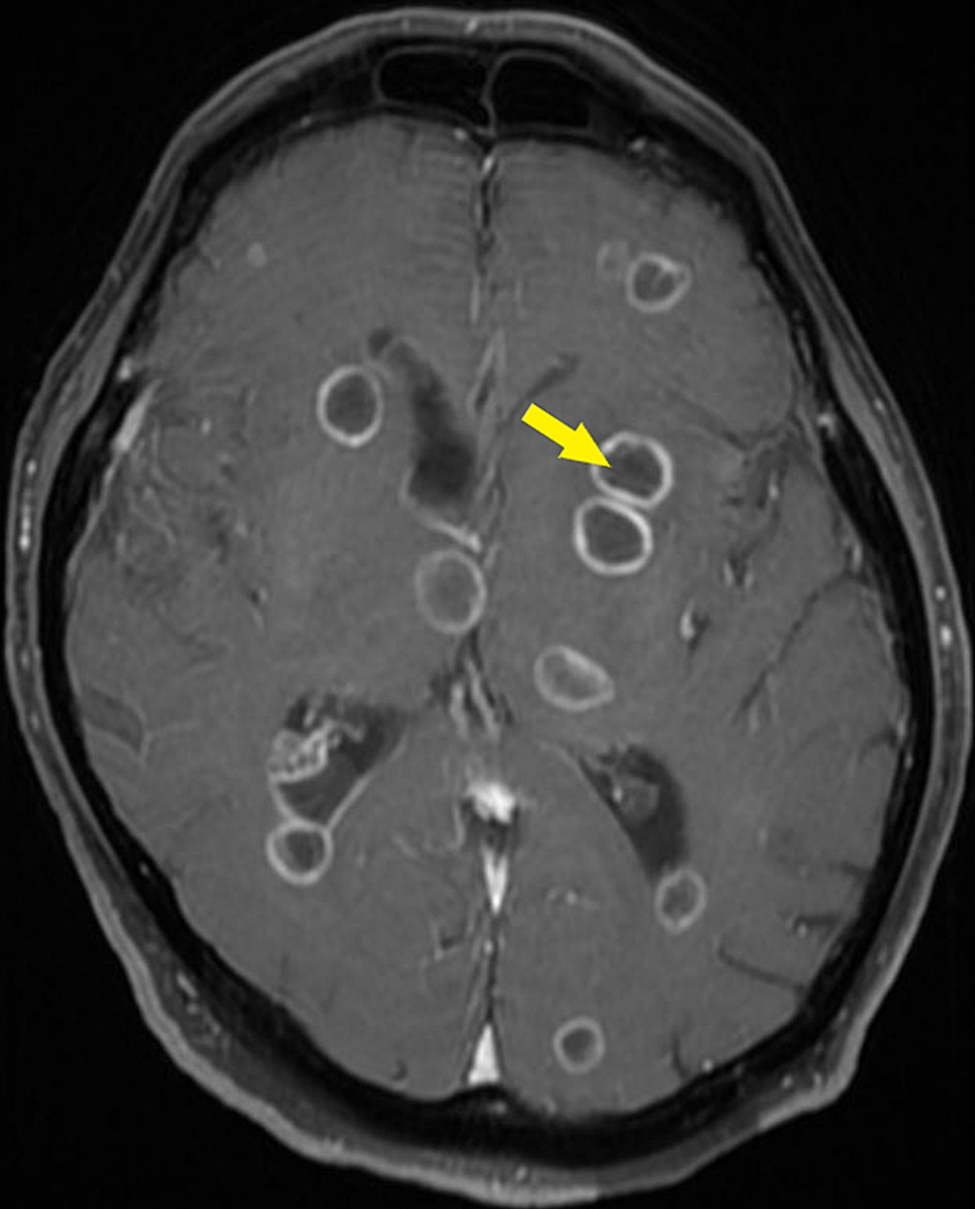
Magnetic resonance imaging showing innumerable parenchymal brain abscesses

Due to progressive encephalopathy, a magnetic resonance imaging (MRI) of the brain with and without contrast was done and revealed innumerable ring-enhancing lesions involving the cerebral hemispheres bilaterally as well as the cerebellar hemispheres (Figure 2). The largest lesion was found in the left temporal lobe and measured 1.6 cm in diameter. There was vasogenic edema surrounding most of the lesions. He was placed on levetiracetam 500 mg every 12 hours for seizure prophylaxis. 

Given *F. nucleatum’*s tendency to cause gingival infections, and the patient’s poor dental hygiene, a maxillofacial CT with contrast was obtained. This showed poor dentition with many maxillary and mandibular carious lesions. Over the course of his eight-day hospital stay, the patient developed rapid metabolic encephalopathy and progressive respiratory failure that required bilevel positive airway pressure support. The patient was transitioned to comfort care and expired on day 8 of admission.

## Discussion

*F. nucleatum* is a non-motile gram-negative anaerobic bacillus that is normally present in the oropharyngeal, upper respiratory, gastrointestinal, and urogenital tracts. It is a rare cause of liver abscesses and intracerebral infections.* F. necrophorum* is known for its association with Lemierre’s syndrome, a rare oropharyngeal infectious disease-causing septic thrombophlebitis of the internal jugular vein seen mostly in young immunocompromised patients [6]. Bacteremia with *Fusobacterium* spp. accounts for less than 1% of all bacteremia and less than 10% for all anaerobic bacteremia cases in adults [7]. The majority of the infections caused by *F. nucleatum *occur in older patients with multiple chronic medical conditions. While disseminated *Fusobacterium *infections spreading to the brain, liver, and other organs have been reported, they are not a common occurrence [7].

The pathogenesis of hepatic infection from *Fusobacterium *is unclear but there are multiple theories. It could either be due to hematogenous spread or spread through the portal circulation. Hematogenous spread can occur from oral sources such as dental caries or peritonsillar abscesses, while spread through the portal circulation could happen secondary to any gastrointestinal illness [8]. However, almost a third of hepatic infections have an unknown cause [9]. Our patient had poor dentition with multiple oral and dental lesions. He has no diagnosed immunosuppression but has a significant history of tobacco and alcohol use disorder. His ultrasound-guided percutaneous drain of the left hepatic abscess showed *Fusobacterium-*infected hepatic abscesses and an MRI of his brain showed multiple ring-enhancing lesions involving both cerebral hemispheres and cerebellar hemispheres. Cerebral infections caused by* F. nucleatum* are rare and are usually associated with underlying causes including periodontal disease, sinusitis, pulmonary infections, intraabdominal infections, gynecologic infections and drug abuse [9]. Based on physical findings as well as the maxillary imaging, we believe that odontogenic infection is the most likely source.

Our patient also had negative blood cultures but yielded the organism by culturing the liver abscess drainage. The sensitivity of blood cultures may be poor especially in those presenting with abscesses. Several reported cases utilized molecular diagnosis for *Fusobacterium* spp. detection. While polymerase chain reaction (PCR) analysis via nucleic acid amplification technique is not regularly performed in most hospitals, it has helped identification in some cases with negative cultures [9,10]. Identification of the pathogen is important for proper antibiotic management.

Prompt and effective antibiotic management is important to improve prognosis [5]. They are typically susceptible to penicillin, clindamycin and metronidazole. Surgical drainage may help with the bacterial burden in most cases of abscesses but was only achieved in the liver lesion for our patient while the innumerable brain lesions were left for conservative management. A previous review of patients with *F. nucleatum* brain abscesses reported surgery in nine of 11 cases. Survival was 100% with more than half of the patients eventually achieving total recovery while five of the 11 had some residual neurological deficits [7]. One fatal case has been described in a young intravenous drug user with *F. nucleatum* abscess involvement of the cerebellum [11]. This case adds to previous reports of *F. nucleatum *infections yet unusual due to extent of infection with both liver and brain involvement resulting in the patient's demise.

## Conclusions

In conclusion, *Fusobacterium* spp. infection should be considered despite being a rare cause of liver and intracerebral infections. Odontogenic infections are predisposing factors even to those with no known immunosuppression. Unfortunately, a presentation can be severe with a high risk for mortality.
